# Effects of Surface-Deacetylated Chitin Nanofibers in an Experimental Model of Hypercholesterolemia

**DOI:** 10.3390/ijms160817445

**Published:** 2015-07-30

**Authors:** Kazuo Azuma, Tomone Nagae, Takeshi Nagai, Hironori Izawa, Minoru Morimoto, Yusuke Murahata, Tomohiro Osaki, Takeshi Tsuka, Tomohiro Imagawa, Norihiko Ito, Yoshiharu Okamoto, Hiroyuki Saimoto, Shinsuke Ifuku

**Affiliations:** 1 Department of Clinical Veterinary Medicine, Faculty of Agriculture, Tottori University, Tottori 680-8533, Japan; E-Mails: ymurahata@muses.tottori-u.ac.jp (Y.M.); tosaki@muses.tottori-u.ac.jp (T.O.); tsuka@muses.tottori-u.ac.jp (T.T.); imagawat@muses.tottori-u.ac.jp (T.I.); taro@muses.tottori-u.ac.jp (N.I.); yokamoto@muses.tottori-u.ac.jp (Y.O.); 2 Graduate School of Engineering, Tottori University, Tottori 680-8552, Japan; E-Mails: tomonekomame@gmail.com (T.N.); h-izawa@chem.tottori-u.ac.jp (H.I.); saimoto@chem.tottori-u.ac.jp (H.S.); 3 Japan Food Research Laboratories, Tama 206-0025, Japan; E-Mail: nagait@jfrl.or.jp; 4 Division of Instrumental Analysis, Research Center for Bioscience and Technology, Tottori University, Tottori 680-8550, Japan; E-Mail: morimoro@chem.tottori-u.ac.jp

**Keywords:** surface-deacetylated chitin nanofibers, chitin nanofibers, chitosan, hypercholesterolemia, rats

## Abstract

This study evaluated the effects of oral administration of surface-deacetylated chitin nanofibers (SDACNFs) on hypercholesterolemia using an experimental model. All rats were fed a high cholesterol diet with 1% *w*/*w* cholesterol and 0.5% *w*/*w* cholic acid for 28 days. Rats were divided equally into four groups: the control group was administered 0.05% acetic acid dissolved in tap water, and the SDACNF, chitosan (CS), and cellulose nanofiber (CLNF) groups were administered 0.1% CNF, CS, or CLNF dissolved in the tap water, respectively, during the experimental period. Changes in body weight, intake of food and water, and organ weight were measured. Serum blood chemistry and histopathological examination of the liver were performed. Administration of SDACNF did not affect body weight change, food and water intake, or organ weights. Administration of SDACNF and CS decreased the diet-induced increase in serum total cholesterol, chylomicron, very-low-density lipoprotein, and phospholipid levels on day 14. Moreover, oral administration of SDACNFs suppressed the increase of alanine transaminase levels on day 29 and suppressed vacuolar degeneration and accumulation of lipid droplets in liver tissue. These data indicate that SDACNF has potential as a functional food for patients with hypercholesterolemia.

## 1. Introduction

Cardiovascular disease is the leading cause of death and disability [1]. The population at risk for atherosclerotic cardiovascular disease is ever increasing as the obesity epidemic and its complications, including diabetes, hypertension, and dyslipidemia, continue to grow among young adults [1]. To date, several authors have reported that certain foods and nutraceuticals are beneficial in the management of serum cholesterol levels [2,3]. For example, dietary fiber, phytosterols, soy protein, nuts, and red yeast rice are beneficial supplements that decrease the level of low-density lipoprotein (LDL) cholesterol [2]. In addition, the use of policosanol and berberine have been reported for the treatment of hypercholesterolemia [3].

Chitin (β-(1,4)-poly-*N*-acetyl-d-glucosamine) is widely distributed in nature and is the second most abundant polysaccharide after cellulose [4]. Chitin occurs in nature as ordered macrofibrils and is the major structural component in the exoskeleton of crab and shrimp shells and the cell wall of fungi and yeast [5]. As chitin is not readily dissolved in common solvents, it is often converted to its more deacetylated derivative, chitosan (CS: (1,4)-linked 2-amino-deoxy-β-glucan) [6,7,8]. CS is a bioactive cationic polysaccharide with antibacterial, antifungal, antioxidant, antidiabetic, anti-inflammatory, anticancer, and hypocholesterolemic properties [9]. In particular, the hypocholesterolemic effects of CS have been well documented [9,10,11]. Anraku *et al.* investigated the effect of water-soluble CS on indices of oxidative stress in normal human volunteers [10]. Treatment with CS for four weeks significantly decreased plasma glucose level and atherogenic index and increased high density lipoprotein cholesterol (HDL). CS treatment also lowered the ratio of oxidized to reduced albumin and increased total plasma antioxidant activity (TPA). The effects of high and low molecular weight chitosans (HMC; 1000 kDa, LMC; 30 kDa) were examined on oxidative stress and hypercholesterolemia using male six-week-old Wistar Kyoto rats as a normal model (Normal-rats) and spontaneously hypertensive rat/ND mcr-cp (SHP/ND) as a metabolic syndrome model (MS-rats) [11]. In Normal-rats and MS-rats, the ingestion of both HMCs and LMCs over a four-week period resulted in a significant decrease in total body weight and glucose, triglyceride (TG), LDL, and serum creatinine levels. In addition, consumption of both CSs decreased the ratio of oxidized to reduced albumin and increased total plasma antioxidant activity. Ingestion of only HMC in MS-rats over a four-week period resulted in a significant decrease in total cholesterol level.

There are established simple methods for the preparation of chitin nanofibers (CNFs) [12,13,14] and, recently, Surface-deacetylation of chitin nanofibers (SDACNFs) has been reported by Isogai *et al.* [15]. In their study, although the nanofiber surface was transformed into chitosan by deacetylation, the core part was maintained as chitin crystal. Due to the cationic electrostatic repulsive force, the deacetylated chitin was easily disintegrated into nanofibers and the SDACNFs were homogeneously dispersed in water. SDACNFs are advantageous for commercial applications in terms of fibrillation efficiency, production cost, and so on. Moreover, NFs having a highly reactive amino group with high dispersibility were found to be useful for surface modification of NF. More recently, biological activities of SDACNF have been reported [15,16,17]. Orally administered SDACNFs suppressed increases in body and epididymal tissue weight and decreased serum levels of leptin and tumor necrosis factor-α [16]. In the circular excision wound model, SDACNFs induced re-epithelialization and proliferation of fibroblasts and collagen tissue [17].

The aim of this study was to evaluate the effects of oral administration of SDACNFs on hypercholesterolemia using an experimental hypercholesterolemia model. Moreover, the effects of SDACNFs were compared with those of CS and cellulose nanofibers (CLNFs).

## 2. Results and Discussion

### 2.1. Effects of Oral Administration of SDACNFs on Body Weight Change, Food and Water Intake, and Organ Weights

Weight changes during the experimental period are shown in Table 1a. The mean body weight of all groups increased, and there was no difference in the body weight among all the experimental groups. Data on food intake during the experimental period are shown in Table 1b. In the CLNF group, food intake was significantly greater than the control group on days 14 and 21 (*p* < 0.05) and the CS group on day 21 (*p* < 0.05). No significant difference in food intake was observed among the experimental groups on other days. Data on water intake during the experimental period are shown in Table 1c. In the CLNF group, water intake was significantly greater than the control, CNF, and CS groups on days 21 and 28 (*p* < 0.01). While SDACNFs did not affect food and water intake during the experimental period, CLNF did. The reports available regarding the bioactivity of orally administered CLNF are limited. Previously, we found no effect of CLNF on food and water intake in a short-term study (5 days) [16,17], and additional experiments on the long-term effects of CLNF should be performed.

**Table 1:** Effects of oral administration of surface-deacetylated chitin nanofibers (SDACNFs) on body weight change and food and water intake. Each table indicates body weight (**a**), food intake (**b**), and water intake (**c**). Data are presented as mean ± standard error. *: *p* < 0.05 compared with the control and CS groups. **^†^**: *p* < 0.05 compared with the control group. **^‡‡^**: *p* < 0.01 compared with the control, CNFs and CS groups.

	Control	SDACNF	CS	CLNF
(a) Body weight (g)				
day 0	187.2 ± 7.3	186.4 ± 5.8	187.6 ± 5.5	185.9 ± 5.5
day 7	233.5 ± 14.4	231.0 ± 7.9	236.7 ± 7.4	231.4 ± 10.6
day 14	285.5 ± 15.1	282.1 ± 11.0	291.4 ± 10.1	288.3 ± 10.6
day 21	288.1 ± 16.8	300.6 ± 10.3	293.9 ± 12.2	307.2 ± 12.5
day 28	333.3 ± 19.9	316.5 ± 13.2	341.4 ± 16.3	351.1 ± 12.5
(b) Food intake (g/day)				
day 7	22.6 ± 0.7	21.3 ± 1.4	22.6 ± 1.0	21.9 ± 2.2
day 14	29.4 ± 1.2	29.1 ± 1.9	30.5 ± 1.4	30.1 ± 1.4
day 21	28.8 ± 1.0	30.4 ± 1.9	29.1 ± 1.4	31.7 ± 1.7 *
day 28	33.9 ± 1.9	33.4 ± 2.9	33.9 ± 2.2	34.4 ± 1.7
(c) Water intake (g/day)				
day 7	37.9 ± 2.4	37.9 ± 6.2	36.9 ± 3.4	42.1 ± 3.4
day 14	45.8 ± 2.9	46.7 ± 6.0	48.2 ± 4.6	52.8 ± 3.1 **^†^**
day 21	50.3 ± 3.4	52.7 ± 7.7	51.1 ± 2.9	64.3 ± 4.1 **^‡‡^**
day 28	53.3 ± 3.8	55.2 ± 5.8	52.6 ± 3.4	67.2 ± 5.0 **^‡‡^**

The results regarding organ weights on day 29 are shown in Table 2. There was no significant difference in organ weights among the experimental groups. These results indicated that oral administration of SDACNFs did not affect organ weights in this model.

**Table 2:** Effects of oral administration of SDACNFs on organ weights. The table indicates the results of liver, kidney, pancreas, cecum, and abdominal fat tissue. Data are presented as mean ± standard error.

(g)	Control	SDACNF	CS	CLNF
Liver	12.9 ± 1.2	12.5 ± 0.7	12.9 ± 1.2	13.2 ± 1.2
Kidney	2.3 ± 0.2	2.2 ± 0.2	2.2 ± 0.2	2.3 ± 0.2
Pancreas	1.3 ± 0.2	1.2 ± 0.2	1.5 ± 0.2	1.4 ± 0.2
Cecum	7.7 ± 1.7	8.1 ± 0.7	7.9 ± 1.0	8.0 ± 0.5
Abdominal fat tissue	6.5 ± 1.0	6.3 ± 1.4	5.8 ± 1.0	6.6 ± 1.0

### 2.2. Effects of Orally Administered SDACNFs on Serum Chemistry

Serum chemistry data on day 14 are shown in Table 3a. In the SDACNF and CS groups, serum total-cholesterol (T-Cho) levels were significantly lower than that of the control group on day 14 (*p* < 0.01). In the CLNF group, serum T-Cho level was significantly lower than that of the control group on day 14 (*p* < 0.05). In the SDACNF, CS, and CLNF groups, serum TG levels were slightly less than the control group, but the difference was not significant. In the SDACNF group, serum phospholipid (PL) level was significantly decreased compared to the CLNF and control groups (*p* < 0.01).

**Table 3:** Effects of oral administration of SDACNFs on serum chemistry. Each table indicates the results of serum chemistry on day 14 (**a**), and on day 29 (**b**). Data are presented as mean ± standard error. **: *p* < 0.01 compared with the control group. *: *p* < 0.05 compared with the control group. **^†^**: *p* < 0.05 compared with the control and cellulose nanofiber (CLNF) groups. **^‡^**: *p* < 0.05 compared with the CLNF group. dL: deciliter.

	Control	SDACNF	CS	CLNF
(a) day 14				
T-cho (mg/dL)	105.2 ± 21.0	66.5 ± 6.2 **	68.2 ± 7.0 **	83.7 ± 8.2 *
T-TG (mg/dL)	78.7 ± 22.8	68.8 ± 14.9	60.0 ± 5.8	59.7 ± 10.8
PL (mg/dL)	152.0 ± 23.8	110.8 ± 6.5 **^†^**	133.7 ± 10.1	153.2 ± 11.5
(b) day 29				
T-cho (mg/dL)	60.2 ± 18.5	47.6 ± 3.8 **^†^**	58.2 ± 10.6	63.2 ± 11.0
T-TG (mg/dL)	47.7 ± 19.4	44.0 ± 7.0 **^†^**	44.5 ± 10.3	57.7 ± 10.6
PL (mg/dL)	89.8 ± 19.6	75.5 ± 9.6	83.2 ± 12.7	83.0 ± 5.5
ALT (U/L)	40.8 ± 7.2	33.8 ± 2.6 **^‡^**	40.0 ± 7.2	43.2 ± 5.5

The results of serum chemistry on day 29 are shown in Table 3b. In the SDACNF group, serum T-Cho and T-TG levels were significantly decreased compared to that of the CLNF groups. In the SDACNF group, moreover, serum ALT level was significantly less than the CLNF group (*p* < 0.05).

Serum lipid levels on day 29 were lower than those on day 14. The difference might come from the consistency of the fasting. Normally, cholesterol homeostasis is maintained by the absorption of alimentary cholesterol and the endogenous synthesis of cholesterol [18]. In fact, the differences of serum lipids levels were reported by the status of the fasting or non-fasting [19]. To confirm the effects of SDACNF on serum lipids, further study focusing the relationships intakes of SDACNF, the fasting time and serum lipids levels must be performed.

Previously, it was demonstrated that CS decreased serum TG and T-cho levels in the MS-rats [11], and this was likely due to the unique ability of CS to bind lipids and bile acids [20,21,22]. Such binding can promote elimination of fat in the stool, reduce the level of bile acid recycling, and induce hepatic synthesis of new bile acid constituents from cholesterol [20,23]. In this study, SDACNF and CS similarly decreased serum T-Cho levels. Moreover, oral administration of SDACNF suppressed the increase of serum PL level. The transfer of PL is promoted by phospholipid transfer protein (PLTP) [24], and PLTP is widely expressed in organs and cells [25]. High levels of PLTP mRNA have been found in the brain, lungs, and gonads, suggesting specific functions of PLTP in these organs [24]. In addition to promoting transfer of phospholipids from very-low-density lipoprotein (VLDL) and chylomicrons (CM) into high-density lipoprotein (HDL) [26], PLTP contributes to the remodeling of HDL particles. Our results indicated that oral administration of SDACNF may affect the activation of the PLTP, and further studies are needed to address this possibility.

### 2.3. Effects of Oral Administration of SDACNFs on Serum T-Cho Contents

As shown in Table 4, serum CM levels in the SDACNF and CS groups were significantly less than the control group on day 14 (*p* < 0.05). In the SDACNF group, serum VLDL level was significantly less than the CLNF group (*p* < 0.05). No change was observed for LDL and HDL serum levels on day 14 among the experimental groups, and there was no difference in serum CM, VLDL, LDL, and HDL levels among experimental groups on day 29.

**Table 4:** Effects of oral administration of SDACNFs on serum T-Cho contents. Each table summarizes serum T-Cho contents on day 14 (**a**) and day 29 (**b**). Data are presented as mean ± standard error. *: *p* < 0.05 compared with the control group. **^†^**: *p* < 0.05 compared with the control and CLNF groups. dL: deciliter.

mg/dL	Control	SDACNF	CS	CLNF
(a) day 14				
CM	33.9 ± 23.0	18.9 ± 13.4 *	18.5 ± 15.6 *	23.6 ± 15.4
VLDL	76.2 ± 44.9	54.4 ± 32.5 **^†^**	62.9 ± 40.6	77.5 ± 44.9
LDL	25.0 ± 13.2	24.1 ± 12.7	24.8 ± 13.4	29.7 ± 15.1
HDL	34.0 ± 18.2	33.7 ± 17.5	39.9 ± 23.8	32.8 ± 18.0
(b) day 29				
CM	14.6 ± 17.3	7.6 ± 6.2	7.4 ± 8.2	15.2 ± 11.5
VLDL	38.6 ± 27.8	28.8 ± 15.8	23.9 ± 22.1	29.8 ± 17.8
LDL	12.5 ± 6.0	11.6 ± 7.0	10.1 ± 6.0	11.3 ± 6.7
HDL	25.6 ± 14.2	27.5 ± 15.1	30.5 ± 17.8	25.6 ± 15.1

In this study, oral administration of SDACNF and CS equally suppressed the increases of serum CM and VLDL on day 14. Triacylglycerols, PLs, and cholesterols were the predominant dietary lipids. An important step in the intestinal digestion of these lipids is their emulsification with bile salts [27,28,29,30]. A CM acts to transport ingested fat and fat-soluble vitamins, and VLDL acts to transport synthesized glyceride [31]. Our results indicated that one possible mechanism of orally administered SDACNFs may be their binding to lipids like CS. However, no studies have yet examined the binding ability of SDACNFs, and further experiments are necessary.

### 2.4. Effects of Oral Administration of SDACNFs on the Lipid Contents and Pathological Examination in the Liver

Results regarding the lipid contents in the liver are shown in Table 5. No difference was observed in the contents of T-Cho, T-TG and PL in the liver.

**Table 5:** Effects of oral administration of SDACNFs on the lipid content in the liver. The table indicates the results of lipid contents in the liver. Data present mean ± standard error.

mg/g	Control	SDACNF	CS	CLNF
T-cho	17.8 ± 3.8	14.6 ± 3.4	15.5 ± 3.1	16.6 ± 2.4
T-TG	32.6 ± 12.7	26.0 ± 3.4	38.1 ± 19.0	30.0 ± 6.2
PL	17.3 ± 4.8	15.0 ± 1.9	13.0 ± 2.9	13.4 ± 4.8

Consistent with previous studies that demonstrated the suppressive effects of CS and SDACNF on lipid accumulation in the liver [5,32], we found that lipid accumulation in SDACNF treated rats was markedly less than in the control [5]. However, no change was observed in this study. One possible reason is liver lipid accumulation in the control group is not severe (Data not shown). To evaluate the effects of SDACNF on liver lipid accumulation, further study using another experimental model must be performed.

## 3. Experimental Section

### 3.1. Animals and Reagents

The animal study was carried out at the Japan Food Research Laboratories (Tokyo, Japan), and experiments were conducted in compliance with the guidelines of the Japanese Association for Laboratory Animal Science. Male SD rats (6 weeks of age; Japan SLC, Inc., Shizuoka, Japan) were housed in a temperature-controlled room (23 ± 2 °C) with a standard 12 h light/dark cycle for one week. They had free access to water and normal diet (Labo MR Stock; Nosan Corporation, Kanagawa, Japan) during acclimation. During the experimental period, rats were individually housed. Chitin (Chitin TC-L, Koyo Chemical. Co., Ltd., Tokyo, Japan) and chitosan (Koyo Chitosan FH-80, Koyo Chemical. Co., Ltd., Tokyo, Japan) powder from crab shells were purchased from Koyo Chemical. CLNFs were purchased from Sugino Machine Co., Ltd. (BiNFi-s cellulose, WMa-10002, Sugino Macine Limited, Uodu, Japan). Concentrations of all samples used in this study were 1.0 wt % in 0.5 wt % aqueous acetic acid.

### 3.2. Preparation of SDACNFs

SDCNFs were prepared by referring to previously reported procedure and modied [15]. Chitin powder (40.0 g) was treated with 20% (*w*/*w*) NaOH (3.0 L) for 6 h under reflux and an argon atmosphere. After deacetylation, the supernatant was removed by decantation, and the precipitate was thoroughly washed with distilled water and 0.5 wt % of aqueous acetic acid by centrifuge to remove some water soluble products of NaOH, AcONa, and alkaline hydrolysed chitin. For mechanical disintegration, the deacetylated chitin was dispersed in 4.0 L of aqueous acetic acid. The sample was passed through a grinder (MKCA6-3; Masuko Sangyo Co., Ltd., Kawaguchi, Japan) at 1500 rpm three times. The concentration, yield and degree of deacetylation of the surface-deacetylated chitin NFs were 0.71%, 74%, and 20 wt %, respectively.

### 3.3. Study Design

The rats were randomized into four groups (*n* = 6 for each group): the control group was administered acetic acid; the SDACNF group was administered CNF (*n* = 6); the CS group was administered CS (*n* = 6); and the CLNF group was administered CLNF (*n* = 6). All rats were fed a normal diet with 1% *w*/*w* cholesterol and 0.5% *w*/*w* cholic acid for 28 days (from days 0 to 28). The control group was administered 0.05% acetic acid dissolved in tap water during the experimental period. The SDACNF, CS, and CLNF groups were administered 0.1% CNF, CS, or CLNF, respectively, dissolved in the tap water during the experimental period. Body weight of each rat was measured on days 0, 7, 14, 21, and 28. The food intake and volume of water intake were measured on days 7, 14, 21, and 28. Blood sampling was done on day 14 without fasting. After a 12-h fast, blood samples were collected and the weight of the liver, kidney, pancreas, cecum (without contents), and abdominal fat tissue (mesentery, kidney, and epidydimal fat tissue) were measured on day 29.

### 3.4. Serum Chemical Analysis

Serum T-Cho, T-TG, and PL levels were determined by the Cho E-test, TG E-test, and PL C-test (Wako Pure Chemical Industries, Ltd., Osaka, Japan), respectively. Serum ALT level was measured using a blood chemical auto analyzer (Dry-Chem 7000; Fujifilm Inc., Tokyo, Japan).

Plasma lipoproteins were analyzed using an on-line dual enzymatic method for simultaneous quantification of total cholesterol, free cholesterol, PLs, and TGs by high-performance liquid chromatography at Skylight Biotech (Akita, Japan), according to a previously described procedure [33,34].

### 3.5. Measurements of the Lipid Contents and Pathological Examination in the Liver

To determine liver T-Cho, T-TG, and PL contents, lipids were extracted from tissues with chloroform/methanol by the Folch method [35] and measured using the Cho E-test, TG E-test, and PL C-test, respectively.

### 3.6. Statistical Analysis

Statistical analyses were performed using Student’s *t*-test and compare the control group. The data are presented as the mean ± standard deviation; *p* < 0.05 was considered a statistically significant difference.

## 4. Conclusions

The present study indicated that oral administration of SDACNF suppressed the increase in serum total cholesterol, chylomicron, VLDL, and PL levels on day 14. Moreover, oral administration of SDACNFs suppressed the increase of alanine transaminase levels on day 29 and suppressed vacuolar degeneration and accumulation of lipid droplets in liver tissue. These data indicate that SDACNF may be a potential functional food for patients with hypercholesterolemia.

## References

[1] McNamara D.J. (2000). Dietary cholesterol and atherosclerosis. Biochim. Biophys. Acta.

[2] Nijjar P.S., Burke F.M., Bloesch A., Rader D.J. (2010). Role of dietary supplements in lowering low-density lipoprotein cholesterol: A review. J. Clin. Lipidol..

[3] Mannarino M.R., Ministrini S., Pirro M. (2014). Nutraceuticals for the treatment of hypercholesterolemia. Eur. J. Intern. Med..

[4] Muzzarelli R.A.A., Gupta N.S. (2011). Chitin nanostructures in living organisms. Chitin: Formation and Diagenesis.

[5] Azuma K., Ifuku S., Osaki T., Okamoto Y., Minami S. (2014). Preparation and biomedical applications of chitin and chitosan nanofibers. J. Biomed. Nanotechnol..

[6] Kurita K. (2001). Controlled functionalization of the polysaccharide chitin. Prog. Polym. Sci..

[7] Rinaudo M. (2006). Chitin and chitosan: Properties and applications. Prog. Polym. Sci..

[8] Pillai K.S., Paul W., Sharma C.P. (2009). Chitin and chitosan polymers: Chemistry, solubility and fiber formation. Prog. Polym. Sci..

[9] Kerch G. (2015). The potential of chitosan and its derivatives in prevention and treatment of age-related diseases. Mar. Drugs.

[10] Anraku M., Fujii T., Furutani N., Kadowaki D., Maruyama T., Otagiri M., Gebicki J.M., Tomida H. (2009). Antioxidant effects of a dietary supplement: Reduction of indices of oxidative stress in normal subjects by water-soluble chitosan. Food Chem. Toxicol..

[11] Anraku M., Michihara A., Yasufuku T., Akasaki K., Tsuchiya D., Nishio H., Maruyama T., Otagiri M., Maezaki Y., Kondo Y. (2010). The antioxidative and antilipidemic effects of different molecular weight chitosans in metabolic syndrome model rats. Biol. Pharm. Bull..

[12] Ifuku S., Nogi M., Abe K., Yoshioka M., Morimoto M., Saimoto H., Yano H. (2009). Preparation of chitin nanofibers with a uniform width as α-chitin from crab shells. Biomacromolecules.

[13] Ifuku S., Saimoto H. (2012). Chitin nanofibers: Preparations, modifications, and applications. Nanoscale.

[14] Ifuku S. (2014). Chitin and chitosan nanofibers: Preparation and chemical modifications. Molecules.

[15] Fan Y., Saito T., Isogai A. (2010). Individual chitin nano-whiskers prepared from partially deacetylated α-chitin by fibril surface cationization. Carbohydr. Polym..

[16] Azuma K., Osaki T., Ifuku S., Maeda H., Morimoto M., Takashima O., Tsuka T., Imagawa T., Okamoto Y., Saimoto H. (2013). Suppressive effects of cellulose nanofibers—Made from adlay and seaweed—On colon inflammation in an inflammatory bowel-disease model. Bioact. Carbohydr. Dietary Fibre.

[17] Azuma K., Osaki T., Ifuku S., Morimoto M., Takashima O., Tsuka T., Imagawa T., Okamoto Y., Saimoto H., Minami S. (2014). Anti-inflammatory effects of cellulose nanofiber made from pear in inflammatory bowel disease model. Bioact. Carbohydr. Dietary Fibre.

[18] Järvisalo M., Raitakari O., Gylling H., Miettinen T.A. (2006). Cholesterol absorption and synthesis in children with type 1 diabetes. Diabetes Care.

[19] Schwab K.O., Doerfe J., Naeke A., Rohrer T., Wiemann D., Marg W., Hofer S.E., Holl R.W. (2009). Influence of food intake, age, gender, HbA1c, and BMI levels on plasma cholesterol in 29,979 children and adolescents with type 1 diabetes—Reference data from the German diabetes documentation and quality management system (DPV). Pediatr. Diabetes.

[20] Ranaldi G., Marigliano I., Vespignani I., Perozzi G., Sambuy Y. (2002). The effect of chitosan and other polycations on tight junction permeability in the human intestinal Caco-2 cell line^1^. J. Nutr. Biochem..

[21] Ormrod D.J., Holmes C.C., Miller T.E. (1998). Dietary chitosan inhibits hypercholesterolaemia and atherogenesis in the apolipoprotein E-deficient mouse model of atherosclerosis. Atherosclerosis.

[22] Gallaher C.M., Munion J., Hesslink R., Wise J., Gallaher D.D. (2000). Cholesterol reduction by glucomannan and chitosan is mediated by changes in cholesterol absorption and bile acid and fat excretion in rats. J. Nutr..

[23] Sugano M., Fujikawa T., Hiratsuji Y., Nakashima K., Fukuda N., Hasegawa Y. (1980). A novel use of chitosan as a hypocholesterolemic agent in rats. Am. J. Clin. Nutr..

[24] Yazdanyar A., Yeang C., Jiang X.C. (2011). Role of phospholipid transfer protein in high-density lipoprotein- mediated reverse cholesterol transport. Curr. Atheroscler. Rep..

[25] Day J.R., Albers J.J., Lofton-Day C.E., Gilbert T.L., Ching A.F., Grant F.J., O’Hara P.J., Marcovina S.M., Adolphson J.L. (1994). Complete cDNA encoding human phospholipid transfer protein from human endothelial cells. J. Biol. Chem..

[26] Jiang X.C., Bruce C., Mar J., Lin M., Ji Y., Francone O.L., Tall A.R. (1999). Targeted mutation of plasma phospholipid transfer protein gene markedly reduces high-density lipoprotein levels. J. Clin. Investig..

[27] Mu H., Hoy C.E. (2004). The digestion of dietary triacylglycerols. Prog. Lipid Res..

[28] Phan C.T., Tso P. (2001). Intestinal lipid absorption and transport. Front. Biosci..

[29] Iqbal J., Hussain M.M. (2009). Intestinal lipid absorption. Am. J. Physiol. Endocrinol. Metab..

[30] Pan X., Hussain M.M. (2012). Gut triglyceride production. Biochim. Biophys. Acta.

[31] Randolph G.J., Miller N.E. (2014). Lymphatic transport of high-density lipoproteins and chylomicrons. J. Clin. Investig..

[32] Seiva F.R., Chuffa L.G., Braga C.P., Amorim J.P., Fernandes A.A. (2012). Quercetin ameliorates glucose and lipid metabolism and improves antioxidant status in postnatally monosodium glutamate induced metabolic alterations. Food Chem. Toxicol..

[33] Usui S., Hara Y., Hosaki S., Okazaki M. (2002). A new on-line dual enzymatic method for simultaneous quantification of cholesterol and triglycerides in lipoproteins by HPLC. J. Lipid Res..

[34] Mizutani H., Sako T., Arai N., Kuriyama K., Yoshimura I., Mori A., Iwase K., Hirose H. (2010). Application of gel permeation HPLC for lipoprotein profiling in dogs. J. Vet. Med. Sci..

[35] Folch J., Lees M., Stanley G.H.S. (1957). A simple method for the isolation and purification of total lipides from animal tissues. J. Biol. Chem..

